# Aversion Encoding and Behavioral State Modulation of Physiologically Defined Cell Types in the Lateral Habenula

**DOI:** 10.1111/ejn.70302

**Published:** 2025-11-24

**Authors:** Ioannis S. Zouridis, Lisa Schmors, Salvatore Lecca, Mauro Congiu, Manuel Mameli, Philipp Berens, Fabio Monteiro, Patricia Preston‐Ferrer, Andrea Burgalossi

**Affiliations:** ^1^ Institute of Neurobiology Eberhard Karls University of Tübingen Tübingen Germany; ^2^ Werner‐Reichardt Centre for Integrative Neuroscience Tübingen Germany; ^3^ Graduate Training Centre of Neuroscience – International Max‐Planck Research School (IMPRS) Tübingen Germany; ^4^ Hertie Institute for AI in Brain Health University of Tübingen Tübingen Germany; ^5^ Department of Fundamental Neuroscience University of Lausanne Lausanne Switzerland; ^6^ Inserm UMR‐S 839 Paris France; ^7^ Tübingen AI Center University of Tübingen Tübingen Germany

**Keywords:** aversion, behavioral state, cell types, clustering, Gaussian mixtures, in vivo electrophysiology, juxtacellular recordings, lateral habenula, neuronal morphology, principal cell diversity

## Abstract

The lateral habenula (LHb) integrates aversive information to regulate motivated behaviors. Despite recent advances in identifying neuronal diversity at the molecular level, in vivo electrophysiological diversity of LHb neurons remains poorly understood. Understanding this diversity is essential for deciphering how information is processed in the LHb. To address this gap, we conducted in vivo juxtacellular recording and labeling of single LHb neurons in mice. Morphological analysis revealed a direct axonal projection of LHb neurons to the mediodorsal thalamus. To analyze in vivo LHb firing patterns, we applied an unsupervised clustering algorithm. This analysis identified four distinct spontaneous firing patterns of LHb neurons, which were consistent across both anesthetized and awake states. To determine whether these firing patterns correlate with function, we recorded neuronal responses to foot shock stimulation in anesthetized mice and monitored spontaneous behavior in awake mice. We found that low‐firing, bursting neurons were preferentially modulated by foot shocks in anesthetized mice and also tracked behavioral states in awake mice. Collectively, our findings indicate significant electrophysiological diversity among LHb neurons, which is associated with their modulation by aversive stimuli and behavioral state.

## Graphical abstract

The lateral habenula is a brain region that integrates information related to aversive stimuli, yet its neuronal diversity remains poorly understood. Using in vivo juxtacellular recordings in mice and unsupervised clustering of electrophysiological features, we identified four distinct, state‐independent firing patterns of LHb neurons. Among these, low‐firing, bursting neurons were preferentially modulated by foot shocks under anesthesia and tracked and behavioral states in awake mice.

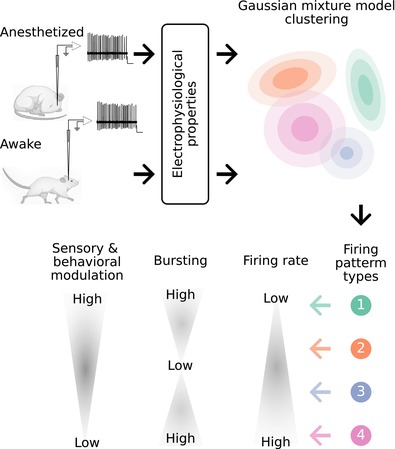

**Abbreviations**
ACG: autocorrelogram
BIC: Bayesian information criterion
BSMI: behavioral state modulation index
DL: dorsolateral
DM: dorsomedial
FS: foot shock
FSMI: foot shock modulation index
GMM: gaussian mixture model
ISI: interspike interval
LHb: lateral habenula
MD: mediodorsal
PC: principal component
sPCA: sparse principal component analysis
VL: ventrolateral
VM: ventromedial

## 1 Introduction

The lateral habenula (LHb) is a bilateral epithalamic structure that integrates signals associated with aversive experiences to regulate motivated behaviors (Hu et al. 2020; Mondoloni et al. 2022). The LHb was initially thought to be a homogeneous brain structure comprised almost exclusively of excitatory, glutamatergic neurons (Aizawa et al. 2012). However, recent work has revealed a high degree of heterogeneity among LHb neurons with respect to their morphological, physiological, molecular, and connectivity properties (Congiu et al. 2023; Hashikawa et al. 2020; Wagner et al. 2017; Wallace et al. 2020; Weiss and Veh 2011). Understanding this diversity is essential for deciphering how LHb integrates information to influence behavioral states and behavior (Michel et al. 2024).

Ex vivo studies have classified LHb neurons into several morphological and electrophysiological subtypes (Wagner et al. 2017; Weiss and Veh 2011). The LHb is organized into hodologically distinct medial and lateral territories, with the medial territory receiving projections from the bed nucleus of the stria terminalis and the lateral territory from the entopeduncular nucleus, each holding segregated projections to the ventral tegmental area and the rostromedial tegmental nucleus (Li et al. 2011; Maroteaux and Mameli 2012; Michel et al. 2024; Shabel et al. 2012). The progress in omics technologies has confirmed this diversity at the molecular level (Hashikawa et al. 2020; Wallace et al. 2020). Moreover, transcriptionally defined LHb subtypes have been shown to have distinct connectivity, indicating a modular network architecture (Wallace et al. 2020). The existence of substantial diversity among LHb neurons is further supported by in vivo observations of diverse spiking patterns as well as variable functional properties; for example, different LHb units respond differently to aversive stimuli and engage differently in learning (Congiu et al. 2019; Lecca et al. 2017). However, the diversity of in vivo activity patterns and how they relate to the encoding of aversive stimuli and behavior remains poorly understood.

In this study, we aimed to fill this gap by clustering in vivo electrophysiological data to identify and quantify the diverse firing patterns of LHb neurons in both anesthetized and awake mice. Moreover, we related spontaneous firing patterns to responses to aversive stimuli and spontaneous behavioral state fluctuations. By resolving the diversity of LHb neurons and examining how this relates to their functional responses, we aim to provide a more accurate and detailed classification of LHb neurons and their modulation by aversive stimuli and behavioral states.

## 2 Material and Methods

### 2.1 Experimental Animals

Experiments were performed on adult male C57BL/6J mice (> 8 weeks old, 20–30 g of body weight; Charles River, Sulzfeld, Germany). The mice were housed under a 12‐h light cycle and were provided with unrestricted access to food and water. All experimental procedures were approved by local authorities and were performed according to the guidelines of the respective local ethics committee: the canton of Vaud Cantonal Veterinary Office Committee for Animal Experimentation (Switzerland), in compliance with the Swiss National Institutional Guidelines on animal experimentation; the Regierungspräsidium Tübingen (Germany) in compliance with the German Animal Welfare Act (TierSchG) and the Animal Welfare Laboratory Animal Ordinance (TierSchVersV).

### 2.2 In Vivo Juxtacellular Recording and Labeling

In vivo electrophysiological recordings of LHb neurons in anesthetized mice have been compiled from previous studies (Congiu et al. 2023; Congiu et al. 2019; Lecca et al. 2017). The procedures and datasets are described in detail in (Congiu et al. 2023). Briefly, the data include juxtacellular recordings of spontaneous activity of LHb neurons in mice anesthetized with either a ketamine‐xylazine mixture or isoflurane. In a subset of experiments (*n* = 64), foot shock (FS) stimulation (1 mA amplitude, 0.5 s stimulus duration, repeated every 5 s, with on average 69 ± 44 trials per neuron) was applied to the contralateral hind limb.

In vivo electrophysiological recordings of LHb neurons in awake head‐fixed mice were performed as previously described (Balsamo et al. 2022; Blanco‐Hernandez et al. 2024). Briefly, mice (*n* = 10) were anesthetized with a ketamine‐xylazine mixture (80–100 and 10 mg/kg, respectively, i.p.), fixed on a stereotaxic frame (Narishige, Tokyo, Japan), and a custom‐made head‐post was attached to the skull using a light‐curing adhesive (Optibond Universal, Kerr) and dental acrylic (Paladur, Heraeus Kulzer, Hanau, Germany). For targeting the LHb, a craniotomy was performed at the stereotactic coordinates: 1.5 mm posterior and 0.5 mm lateral to bregma. During the intervals between recording sessions, the craniotomy site was covered with a silicon sealant (Kwik‐Cast, WPI). Neurobiotin‐filled (1.5–2% Neurobiotin, SP‐1120, Vector Laboratories in “intracellular‐like” solution (in mM: 135 K‐gluconate, 10 HEPES, 10 Na_2_‐phosphocreatine, 4 KCl, 4 MgATP, and 0.3 Na3GTP; osmolarity: 280–310 mOsm) glass pipette electrode [Cat#1403547, Hilgenberg, Malsfeld, Germany], pulled to a 7‐ to 9 MΩ resistance with a micropipette puller (Cat#P‐1000, Sutter Instrument, Novato, CA, USA) was lowered into the LHb (coordinates: 2.3‐ to 2.9 mm ventral to the brain surface). Juxtacellular voltage signals were acquired using an ELC‐03XS amplifier (NPI Electronic, Tamm, Germany) and digitized at 25 kHz (Power1401‐3, Spike2 v8.02; CED, Cambridge, UK). Upon establishing juxtacellular configuration with a neuron, spontaneous spiking activity was monitored. When feasible, once the recording was completed, neurons were labeled by delivering repetitive, square current pulses (200 ms pulse duration, 50% duty charge) (Pinault 1996). Recordings from a session were included in the analysis only if histologically retrieved cells or electrode tracks (in the case of awake recordings) were confirmed to be within the LHb.

### 2.3 Histology and Morphological Reconstructions

Following juxtacellular labeling, animals were overdosed with pentobarbital (300 mg/kg, i.p.), transcardially perfused (0.1 M phosphate buffer followed by a 4% paraformaldehyde 0.1 M phosphate buffer solution), and overnight incubated in fixative. The brain tissue was processed essentially as previously described in Zouridis et al. (2024). Briefly, serial tissue sections (70 μm thickness; coronal or parasaggital sectioning plane) were obtained using a vibrating blade microtome (VT1200, Leica, Germany), washed in PBS, permeabilized with 0.1% Triton in PBS, and incubated successively with a fluorescent streptavidin conjugate (1:1000, Cat#S11225, Thermo Fisher Scientific, overnight at 4°C) and DAPI (1:1000, Cat#D1306, Thermo Fisher Scientific), and imaged using epifluorescence microscopy (Axio Imager, Carl Zeiss, Germany). To reveal the morphology of juxtacellularly labeled cells (i.e., filled with neurobiotin), fluorescent staining was then converted into a dark DAB reaction product essentially following the procedures reported in (Balsamo et al. 2022; Preston‐Ferrer et al. 2016). Local axonal morphologies were inspected in the subset of neurons with high‐quality fillings (*n* = 41), as indicated by strong DAB staining and the presence of a long‐range axon projecting through the fasciculus retroflexus and reaching ventral midbrain regions (not shown); of these, ~̴̴ 39% (16 out of 41) displayed an axonal projection to the mediodorsal (MD) thalamus. Somatodendritic domains (and part of the axonal domains) were reconstructed manually from DAB‐converted tissue sections using Neurolucida (MBF Bioscience, v. 2022.2.3). Dendritic number, length, node count, and maximum branching order were quantified using Neurolucida. Tissue shrinkage correction was performed in the Z dimension. We acknowledge that the observations regarding axonal bouton morphologies are derived from a light microscopy approach. Although previous studies have demonstrated a strong correspondence between light microscopy and ultrastructural analyses (Jinno et al. 2007; Klausberger et al. 2003; Sadek et al. 2007), future electron microscopy investigations will be necessary to further validate our observations.

### 2.4 Analysis of Electrophysiological and Behavioral Data

The analysis of juxtacellular electrophysiological data was performed as previously described (e.g., Zouridis et al. 2024). Specifically, spikes were isolated from continuous voltage traces using a threshold‐based approach and visual inspection. For each neuron, we computed the mean firing rate, the interspike interval (ISI) distribution (range 0–0.5 s), the coefficient of variation (CV; calculated as the ratio of the standard deviation of the ISI over the mean ISI; Softky and Koch 1993), the burst index (calculated as the percentage of ISIs shorter than 25 ms), and the autocorrelogram (ACG; range ± 0.1 s) based on spontaneous activity. Temporal features of the ISI distributions and ACGs were extracted with sparse principal component analysis (sPCA; Zou et al. (2006)) using the first eight principal components (PCs). Recordings were included in the analysis if histological analysis confirmed the recording position in the LHb and the electrophysiological traces showed no signs of cellular damage, such as spike widening and firing rate increase. The resulting dataset consisted of 295 recordings in awake head‐fixed animals and 270 recordings in anesthetized animals from Congiu et al. (2023), 88 of which were originally recorded in Congiu et al. (2019) and 33 in Lecca et al. (2017).

To assess the responses of neurons to FS stimuli, we constructed peristimulus time histograms (−0.2 to +1.3 s with respect to stimulus onset; bin width, 20 ms). To quantify the strength of FS modulation, we computed a FS modulation index (FSMI), defined as follows:

$$\text{FSMI}=\frac{{\mathrm{FR}}_{\text{stimulus}}-{\mathrm{FR}}_{\text{baseline}}}{{\mathrm{FR}}_{\text{stimulus}}+{\mathrm{FR}}_{\text{baseline}}}$$

where FR_baseline_ represents the mean firing rate during 1 s preceding the stimulus onset, FR_stimulus_ represents the mean firing rate during stimulus delivery. Neurons with firing rate < 1 Hz were excluded because calculating the index for these neurons is not feasible due to them potentially generating fewer than one spike per stimulation duration. To quantify the sustained effect of stimulation on neural firing, we computed the area under the curve on the z‐scored responses during the first 300 ms after stimulus onset.

To partition behavior into states, we defined “active” epochs as those with facial motion energy above the 80th percentile and “quiet” epochs below the 20th percentile. To quantify the strength of modulation, we computed a behavioral state modulation index (BSMI), defined as follows:

$$\text{BSMI}=\frac{{\mathrm{FR}}_{\text{active}}-{\mathrm{FR}}_{\text{quiet}}}{{\mathrm{FR}}_{\text{active}}+{\mathrm{FR}}_{\text{quiet}}}$$

where FR_active_ represents the mean firing rate during epochs of “active” behavioral state and FR_quiet_ represents the mean firing rate during epochs of “quiet” behavioral state. For every neuron, we tested the firing rate modulation in “active” versus “quiet” behavioral states using an unpaired Wilcoxon signed‐rank test at a 0.05 significance level. Neurons were categorized into three groups based on whether they exhibited a significant increase, decrease, or no significant firing rate modulation by brain state.

To track facial motion, the mouse's snout was illuminated with infrared light (M850L3, Thorlabs) and video was acquired (DMK33UX265 and IC Capture software, TheImagingSource) via triggering (Power1401–3, Spike2 v8.02; CED, Cambridge, UK). Whisker‐pad motion energy (region of interest including the whisker‐pad) was extracted and analyzed with the open‐source software facemap (Syeda et al. 2024), essentially as described in Blanco‐Hernandez et al. (2024); Stringer et al. (2019).

### 2.5 Clustering Analysis

We fit each data set with a Mixture of Gaussians model (Bishop and Nasrabadi 2006) using the following 19 features: mean FR, CV, burst index, first 8 PCs of the ISI distribution, and first eight PCs of the ACG. We applied the Bayesian Information Criterion (BIC; [38]) to determine the optimal number of clusters: $\mathrm{BIC}=-2\log\left[L\right]+M\;\log\left[N\right]$, with L as the likelihood function for the model and M as the number of parameters in the model. The BIC naturally implements Occam's razor by favoring simpler models (fewer clusters) unless there is strong evidence in the data for more complex models (more clusters). To avoid local minima, we initialized the GMM algorithm 100 times per candidate cluster number and selected the number of clusters with the smallest mean BIC and the point of saturation of its gradient. Regardless of the number of sPCA components, we consistently identified a minimum of four clusters for both datasets. Accounting for the expected low to moderate correlations between features (μ_Anesthetized_ = 0.29, μ_Awake_ = 0.28) had no substantial impact on the clustering results. To assess each feature's contribution to the clustering, we calculated the absolute difference between each cluster's mean and the global mean for that feature. To match clusters from the anesthetized preparation with clusters from the awake preparation, we used the cosine similarity SC (Singhal 2001) to measure the similarity between cluster centroids:

$$S_{C}\left({\text{cluster}}_{A},{\text{cluster}}_{B}\right)=\frac{A\cdot B}{\left\|A\right\|\;\left\|B\right\|}$$

We applied the Hungarian algorithm (Kuhn 1955; Munkres 1957) to find the optimal matching between clusters from the two datasets. It optimally matches clusters by minimizing the total dissimilarity $\min_{\pi \epsilon \Pi }\sum_{i=1}^{n}\left(1-S_{C}\right)$, where *π* is a permutation of cluster indices, and Π is the set of all possible combinations. We further filtered matches based on a similarity threshold of 0.6, ensuring that only cluster pairs with sufficient similarity were included.

### 2.6 Statistical Analysis

Data analysis was performed using custom‐written code based on packages such as numpy (v. 1.22.3), sklearn (v. 1.0.2), matplotlib (v. 3.5.2), seaborn (v. 0.13.2), and pandas (v. 1.4.2). All statistical analyses were conducted using the Python libraries scipy (v. 1.8.0) and statsmodels (v. 0.13.2). For statistical analysis, we perform an unpaired Wilcoxon signed‐rank test at a 0.05 significance level. To compare more than two groups, we used ANOVA with *α* = 0.05 and Tukey's HSD post hoc test. Throughout the manuscript, boxplots show medians (black), quartiles (boxes), and ranges (whiskers) with outliers shown. Values throughout the text are expressed as mean ± SD unless otherwise stated.

## 3 Results

### 3.1 In Vivo Juxtacellular Recordings and Morphological Identification of Single LHb Neurons

**FIGURE 1:**
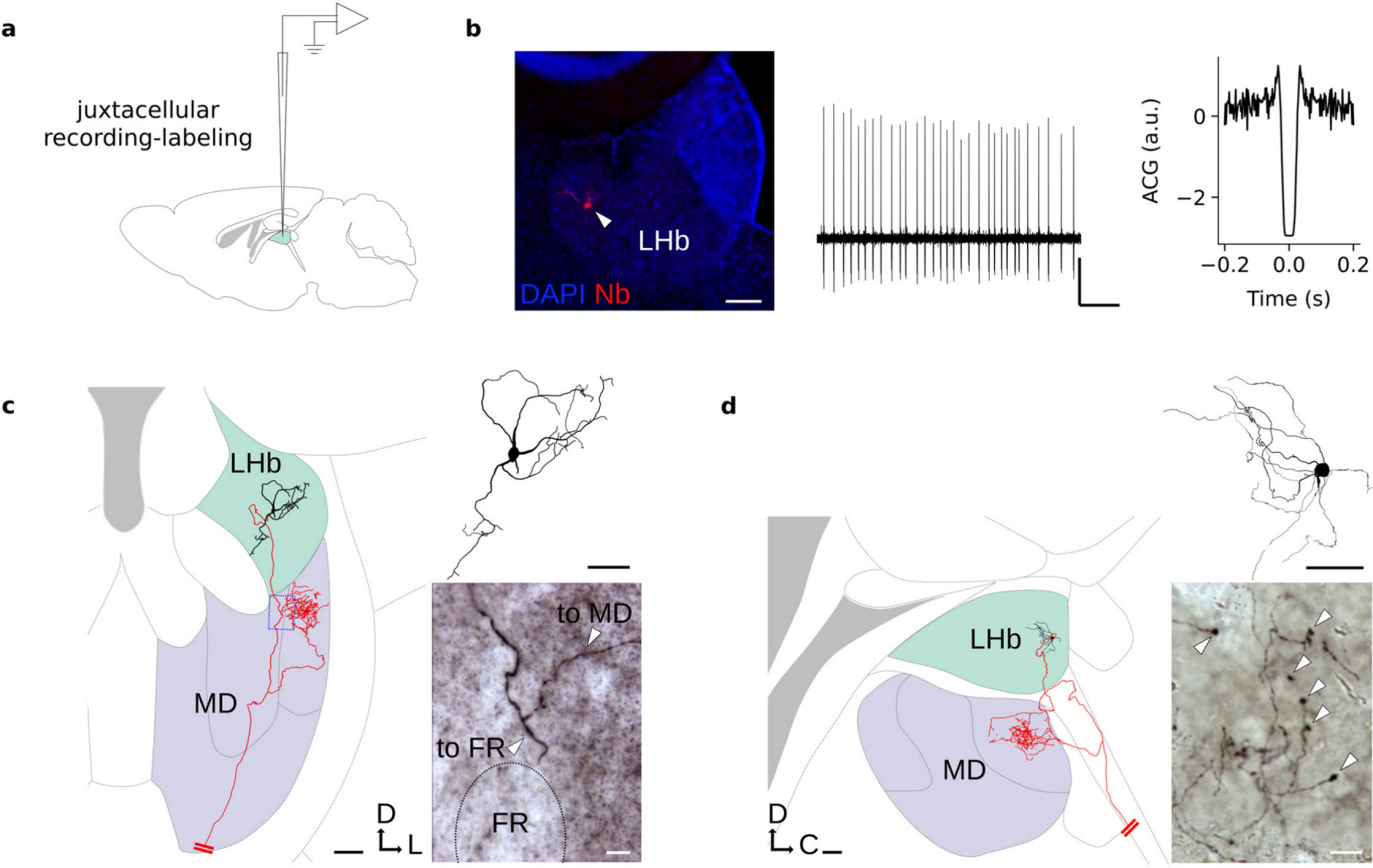
In vivo juxtacellular recording and labeling of individual LHb neurons. (a) Schematic representation of juxtacellular recording‐labeling of LHb neurons. (b) Histological identification (left; soma indicated by arrowhead; scale bar, 100 μm), juxtacellular voltage trace (middle; scale bars, 1 mV, 200 ms), and autocorrelogram (right) of a representative LHb neuron. (c) Morphological reconstruction of a juxtacellularly labeled LHb neuron (black, somatodendritic compartment; red, axon) in a coronal sectioning plane. Neurite thickness has been adjusted for display purposes. Right panel, extended focus projection image showing the axonal branching point towards the mediodorsal (MD) thalamus and the fasciculus retroflexus (arrowheads). Scale bars; left, 100 μm; right top, 50 μm; right bottom, 10 μm. (d) Same as (c), but for a LHb neuron reconstructed in a parasagital sectioning plane. Bottom right, close‐up magnification on axonal boutons within the MD. Large boutons are indicated by arrowheads. Scale bars; left, 100 μm; right top, 50 μm; right bottom, 10 μm. C, caudal; D, dorsal; L, lateral.

To investigate the in vivo activity and morphological characteristics of individual LHb neurons, we employed juxtacellular recording and labeling in anesthetized mice (Figure 1a; see Section 2). This technique enables high signal‐to‐noise recording of single‐neuron activity and also allows for post hoc morphological reconstruction (Figure 1b,c,d). The dendrites of reconstructed LHb neurons were confined within the anatomical boundaries of the LHb (Figure 1c,d). Each neuron emitted a single axon that projected through the fasciculus retroflexus towards the ventral midbrain targets (not shown). Remarkably, in a subset of identified LHb neurons (~ 39%; 16 out of 41 with high‐quality axonal filling; see Section 2), we identified axonal collaterals targeting the MD thalamus. As illustrated in the representative examples (Figure 1c,d), one or two axonal branches extended rostro‐laterally and arborized extensively within the MD nucleus. These projections formed large en passant and terminal boutons (Figure 1d), suggestive of strong synaptic influence.

Together, these data provide direct anatomical evidence for an LHb‐to‐MD thalamus projection, thus providing a pathway through which affective signals may influence high‐order thalamocortical processing.

### 3.2 LHb Neurons Spontaneously Fire in Four Distinct Patterns

**FIGURE 2:**
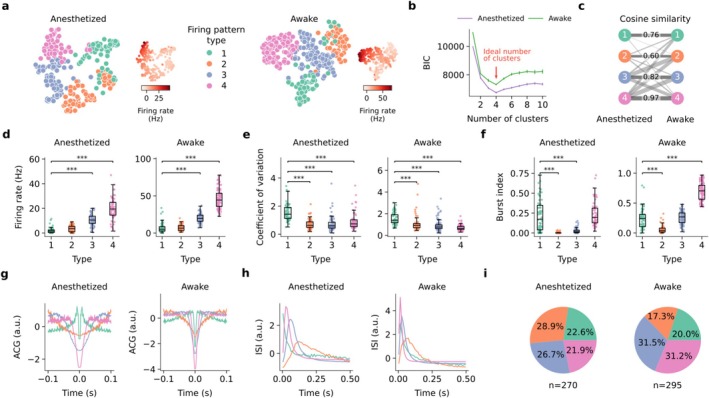
Gaussian mixture modeling of spontaneous firing features identifies four firing pattern types of LHb neurons. (a) t‐SNE embedding based on 19 spontaneous firing features of LHb neurons recorded under anesthesia (left; n_neurons_ = 270) and awake recordings (right; n_neurons_ = 295). Each point represents a recording. Firing pattern types (color‐coded) were determined using Gaussian mixture modeling (GMM) clustering using the same 19 features. Matching colors indicate the corresponding clusters across both conditions (see Section 2). The insets show t‐SNE embedding colored by the mean spontaneous firing rate for each neuron. (b) Bayesian information criterion (BIC) computed for different numbers of clusters. Arrow indicates minimum BIC and the ideal number of clusters. For each number of clusters, the GMM was initialized randomly 100 times and the error bars show the confidence intervals (CI) across runs. (c) Clusters of anesthetized and awake recordings were matched using the Hungarian algorithm. Line widths are proportional to the value of computed cosine similarity. (d) Mean firing rate for each firing pattern type under anesthesia (left) and awake (right). Number of recordings and conventions as in (a) (Anesthetized: ANOVA, *p* < 0.001; Type‐1 vs. Type‐3: mean difference = 8.60, 95% CI [6.25, 10.95], *p* < 0.001; Type‐1 vs. Type‐4: mean difference = 17.50, 95% CI [15.03, 19.96], *p* < 0.001; Awake: ANOVA, *p* < 0.001; Type‐1 vs. Type‐3: mean difference = 13.03, 95% CI [9.23, 16.84], *p* < 0.001; Type‐1 vs. Type‐4: mean difference = 39.28, 95% CI [35.46, 43.09], *p* < 0.001; results of Tukey's HSD). Significance bars with * for *p* < 0.05, ** for *p* < 0.01, and *** for *p* < 0.001. (e) Same as in (d) but for coefficient of variation (Anesthetized: ANOVA, *p* < 0.001; Type‐1 vs. Type‐2: mean difference = −0.82, 95% CI [−1.05, −0.59], *p* < 0.001; Type‐1 vs. Type‐3: mean difference = −0.80, 95% CI [−1.03, −0.56], *p* < 0.001; Type‐1 vs. Type‐4: mean difference = −0.59, 95% CI [−0.84, −0.34], *p* < 0.001; Awake: ANOVA, *p* < 0.001; Type‐1 vs. Type‐2: mean difference = −0.44, 95% CI [−0.70, −0.17], *p* < 0.001; Type‐1 vs. Type‐3: mean difference = −0.64, 95% CI [−0.87, −0.41], *p* < 0.001; Type‐1 vs. Type‐4: mean difference = −0.88, 95% CI [−1.11, −0.65], *p* < 0.001; results of Tukey's HSD) (f) Same as in (d) but for burst index (Anesthetized: ANOVA, *p* < 0.001; Type‐1 vs. Type‐2: mean difference = −0.21, 95% CI [−0.27, −0.16], *p* < 0.001; Type‐1 vs. Type‐3: mean difference = −0.19, 95% CI [−0.24, −0.14], *p* < 0.001; Awake: ANOVA, *p* < 0.001; Type‐1 vs. Type‐2: mean difference = −0.18, 95% CI [−0.24, −0.11], *p* < 0.001; Type‐1 vs. Type‐4: mean difference = 0.45, 95% CI [0.40, 0.51], *p* < 0.001; results of Tukey's HSD) (g, h) Mean autocorrelograms (g) and interspike interval distributions (h) for LHb neurons recorded under anesthesia (left) and awake recordings (right). Number of neurons and conventions as in (a). (i) Proportions of neurons per cluster for the anesthetized (left) and awake (right) conditions.

To explore the diversity of spontaneous firing patterns of LHb neurons, we compiled datasets of in vivo juxtacellular recordings from anesthetized and awake preparations and visualized the firing patterns in two‐dimensional space using t‐distributed Stochastic Neighbor Embedding (t‐SNE, [14]; Figure 2a). Comparative analysis showed that neuronal firing properties were similar between ketamine‐xylazine and isoflurane‐anesthetized mice (Figure S1), enabling us to merge recordings from both anesthetic conditions into a single dataset. As expected, due to ketamine's antagonism of NMDA receptors, which can suppress bursting activity in LHb neurons (Yang et al. 2018), spike bursting was higher under isoflurane anesthesia (Figure S1d) with differences in interspike interval (ISI) distributions and spike ACG observed only during specific time components directly related to bursting (Figure S1e,f). To investigate the number and nature of spontaneous firing patterns of LHb neurons, we used Gaussian mixture model (GMM) clustering on each dataset separately. We used 19 features, including firing rate, burst index, coefficient of variation, and sparse principle component (sPCA) features for ISI and ACG (Figure S2a,b). We used the Bayesian information criterion (BIC) along with the saturation point of its gradient to determine the optimal number of clusters, testing across different numbers of sparse components (Figure S2c). This analysis yielded a minimum of four identified LHb groups in each dataset, referred to as firing Type‐1 through 4 thereafter (Figures 2a,b and S2a–c). Features most influential for identifying clusters were firing rate and spike bursting‐related metrics, such as the “burst index” and specific sparse components of the ISI and ACG associated with spike bursting (see Figure S2d‐i). Accounting for feature co‐linearity did not substantially alter the clustering results (Figure S2f,g). To compare the firing pattern types of LHb neurons across awake and anesthetized states, we measured the cluster similarity by computing the SC and matching them using the Hungarian algorithm (see Section 2; Figure 2c, SC(1) = 0.76, SC(2) = 0.60, SC(3) = 0.82, and SC(4) = 0.97). We identified corresponding firing pattern types with similar properties in both the anesthetized and awake datasets (Figure 2d–h). Although mean firing rates were higher in the awake state (Figures 2d and S3), the direct correspondence of firing pattern types between the datasets and the well‐balanced presence of each type in both conditions (anesthetized: Type‐1: *n* = 61, Type‐2: *n* = 78, Type‐3: *n* = 72, Type‐4: *n* = 59; awake: Type‐1: *n* = 59, Type‐2: *n* = 51, Type‐3: *n* = 93, Type‐4: *n* = 92; Figure 2i) indicates that these firing types are consistent across both states and not exclusive to one.

Type‐1 firing patterns were characterized by a low mean spontaneous firing rate (anesthetized: 2.07 ± 2.29; awake: 7.15 ± 6.78; Figure 2d), high irregularity as indicated by a high coefficient of variation (anesthetized: 1.53 ± 0.58; awake: 1.57 ± 0.57; Figure 2e), and bursty activity as shown by a high mean burst index (anesthetized: 0.22 ± 0.20; awake: 0.24 ± 0.17; Figure 2f) and an early peak in the ACG and ISI distributions (Figure 2g,h). Notably, Type‐1 bursting patterns were recorded under both anesthetic conditions (Type‐1 n_ketamine_ = 15 and n_isoflurane_ = 46; Figure S1l), confirming that these firing patterns represent genuine neuronal properties rather than anesthesia‐induced artifacts. Firing pattern Type‐2 neurons exhibited a low mean spontaneous firing rate (anesthetized: 3.45 ± 2.47 Hz; awake: 7.37 ± 4.23 Hz; Figure 2d), a low burst index (anesthetized: 0.0038 ± 0.0078; awake: 0.06 ± 0.07; Figure 2f), and a tendency to fire regularly, as indicated by the late peaks in ACG and ISI distributions (Figure 2g,h). Type‐3 firing patterns showed a higher mean firing rate (anesthetized: 10.67 ± 4.61; awake: 20.18 ± 5.80; Figure 2d), a moderate burst index (anesthetized: 0.03 ± 0.03; awake: 0.26 ± 0.10; Figure 2f), and a low coefficient of variation (anesthetized: 0.74 ± 0.54; awake: 0.93 ± 0.54; Figure 2e). Type‐4 firing patterns displayed a high firing rate (anesthetized: 19.57 ± 9.25; awake: 46.42 ± 13.32; Figure 2d), a high burst index (anesthetized: 0.24 ± 0.16; awake: 0.69 ± 0.15; Figure 2f), and a low coefficient of variation (anesthetized: 0.95 ± 0.62; awake: 0.69 ± 0.24; Figure 2e). Based on our limited dataset of recordings that could be unambiguously matched to histologically identified neurons, we did not find a clear anatomical organization of the LHb clusters or a consistent relationship between cluster identity and neuronal morphology (Figure S4).

### 3.3 Type‐1 Firing Pattern LHb Neurons Are Preferentially Modulated by Aversive Stimuli

**FIGURE 3:**
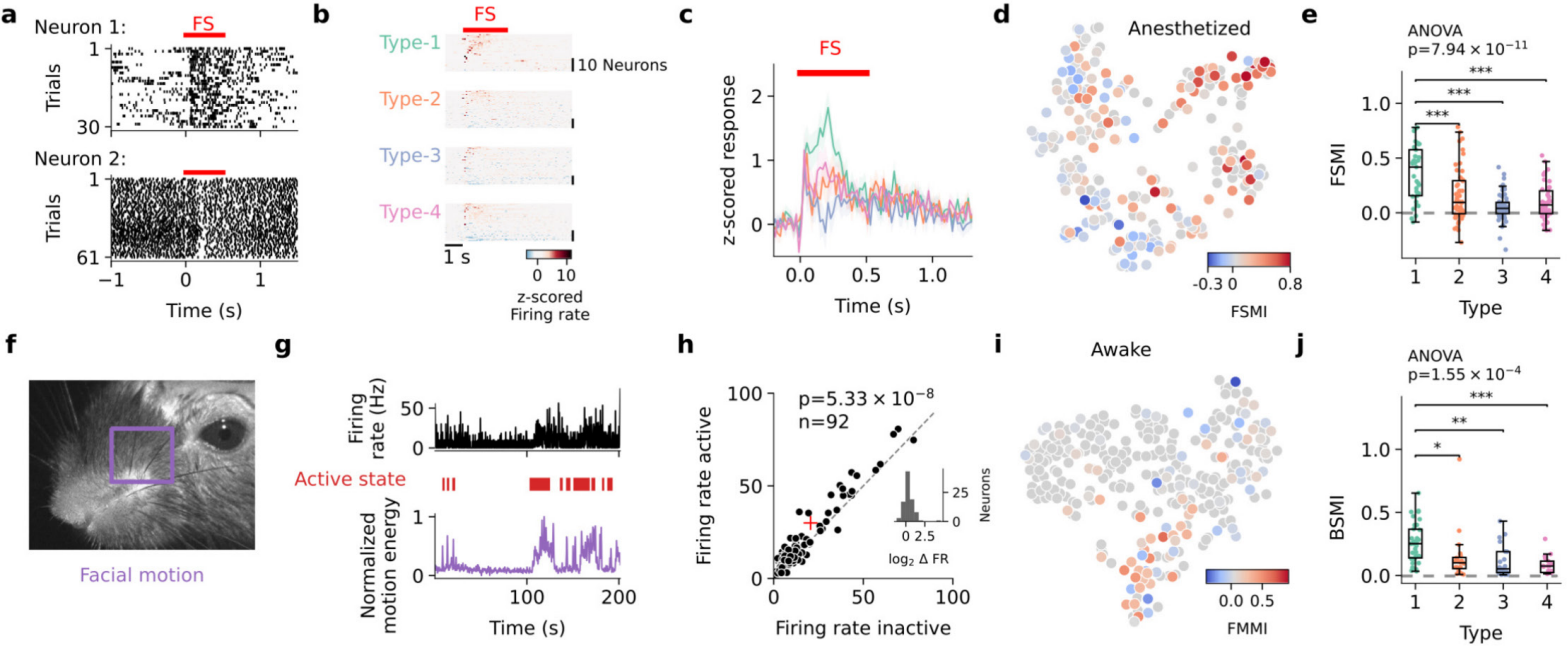
Sensory and behavioral state modulation of LHb neurons across different firing pattern types. (a) Responses to foot shock (FS) stimulation for two representative neurons in a spike raster plot. FS stimulation onset and duration indicated by a red line. (b) FS responses split by type. FS stimulation onset and duration indicated by a red line. (c) Mean FS responses of the four LHb neuronal types. FS stimulation onset and duration indicated by a red line. (d) t‐SNE embedding of spontaneous firing patterns color‐coded by the FS modulation index (n_neurons_ = 219; neurons without FS recordings are shown in gray). (e) Comparison of FS modulation index (FSMI) across different LHb neuronal types (ANOVA, n_neurons_ = 219, *p* = 7.94 × 10^–11^). Firing pattern Type‐1 neurons were more strongly modulated in comparison to other types (Type‐1 vs. Type‐2: mean difference = −0.20, 95% CI [−0.32, −0.09], *p* < 0.001; Type‐1 vs. Type‐3: mean difference = −0.31, 95% CI [−0.42, −0.20], *p* < 0.001; Type‐1 vs. Type‐4: mean difference = −0.26, 95% CI [−0.38, −0.14], *p* < 0.001; Type‐2 vs. Type‐3: mean difference = −0.11, 95% CI [−0.20, −0.01], *p* = 0.0206). Significance bars with * for *p* < 0.05, ** for *p* < 0.01, and *** for *p* < 0.001. (f) Example video frame for facial motion analysis. The square box indicates the region of interest used for extracting whisker‐pad motion energy. (g) Representative recording of a LHb neuron along with orofacial videography. Top: firing rate; middle: active states are marked in red; everything else is quiet immobility; bottom: whisker pad motion energy over time. (h) Effect of behavioral state on LHb neurons' firing rate. P value denotes the result of a paired Wilcoxon signed‐rank test, *n* = 92, mean: 20.58 Hz vs. 29.97 Hz, indicated with red cross. Inset: Histogram of firing rate fold‐change in “active” relative to “quiet” behavioral states (∆ FR log2‐ratio). (i) t‐SNE embedding of awake recordings color‐coded by behavioral state modulation index (n_neurons_ = 92; neurons without facial motion recorded depicted in gray). Of the recorded neurons, 65.2% increase, 19.6% decrease activity, and 15.2% show no change in activity. (j) Absolute behavioral state modulation index (BSMI) across different LHb firing patterns (ANOVA, n_neurons_ = 92, *p* = 1.55 × 10–4). Type‐1 neurons were more strongly modulated in comparison to other types (Type‐1 vs. Type‐2: mean difference = −0.12, 95% CI [−0.23, −0.01], *p* = 0.031; Type‐1 vs. Type‐3: mean difference = −0.15, 95% CI [−0.26, −0.04], *p* = 0.003; Type‐1 vs. Type‐4: mean difference = −0.18, 95% CI [−0.30, −0.06], *p* < 0.001). Significance bars as in (e).

LHb neurons are known to exhibit varied excitatory and inhibitory responses to aversive sensory stimuli, such as FSs (Congiu et al. 2019; Lecca et al. 2017; two example responses are shown in Figure 3a). To investigate how different spontaneous firing pattern types relate to the response to aversive stimuli, we recorded neuronal responses to FS stimulation in anesthetized animals and computed an FSMI (see Section 2). In a previous study, we demonstrated that the FSMI is negatively correlated with the spontaneous mean firing rate of LHb neurons (Congiu et al. 2023). Since neurons of different spontaneous firing types vary in their mean firing rates, we expected to observe differences also in their FS responses. Indeed, we observed varied responses across all four spontaneous firing types, with each type containing both FS‐excited (FSMI > 0) and inhibited (FSMI < 0) neurons. For all firing pattern types, the mean FS responses were dominated by short‐latency excitatory components (Figure 3b,c). Interestingly, neurons with spontaneous firing pattern Type‐1 exhibited, on average, FS responses with a prominent long‐latency excitatory component (response peaking at 210 ms; Figure 3b,c). Further comparison of Type‐1 against other spontaneous firing patterns revealed an on average higher FS modulation in Type‐1 neurons (ANOVA on FSMI, *p* < 0.001 n neurons = 219; Type‐1 vs. Type‐2: mean difference = −0.205, 95% CI [−0.318, −0.091], *p* < 0.001; Type‐1 vs. Type‐3: mean difference = −0.313, 95% CI [−0.423, –0.203], *p* < 0.001; Type‐1 vs. Type‐4: mean difference = −0.262, 95% CI [−0.379, –0.144], *p* < 0.001; Figure 3d,e). These results indicate that Type‐1 neurons are comparatively more strongly and sustainedly modulated by FS stimulation (ANOVA on area under the curve, *p* < 0.001 n_neurons_ = 219; Type‐1 vs. Type‐2: mean difference = −9.49, 95% CI [−16.98, −2.00], *p* < 0.001; Type‐1 vs. Type‐3: mean difference = −13.92, 95% CI [−21.19, −6.65], *p* < 0.001; Type‐1 vs. Type‐4: mean difference = −7.91, 95% CI [−15.66–0.16], *p* < 0.05). Further, FS modulation was positively correlated with burstiness (Pearson *r* = 0.29, *p* = 3.76 × 10^−5^) and negatively correlated with firing rate (Pearson *r* = −0.36, *p* = 4.47 × 10^−7^), both prominent in Type‐1 neurons.

### 3.4 Type‐1 Firing Pattern LHb Neurons Are Preferentially Modulated by Behavioral State

Recent work in awake head‐fixed mice has demonstrated that spontaneous behavioral state fluctuations are accompanied by brain‐wide modulations of neuronal activity (Bimbard et al. 2023; Blanco‐Hernandez et al. 2024; Oude Lohuis et al. 2024; Steinmetz et al. 2019; Stringer et al. 2019). Notably, while this activity modulation appears to be a brain‐wide phenomenon, only a fraction of neurons is significantly modulated within each brain area, pointing to cell‐type‐specific mechanisms. To investigate whether and how the different LHb neuronal types are modulated by behavioral state, we acquired juxtacellular recordings along with orofacial videography in awake head‐fixed mice.

Using facial motion energy to index behavioral states (Blanco‐Hernandez et al. 2024; Stringer et al. 2019), we categorized the animal's behavioral state into “active” and “quiet” (see Section 2; Figure 3f). On average, facial motion was associated with a significant net increase in neuronal activity (Wilcoxon test, n neurons = 92, *p* = 5.33 × 10^–8^, 20.58 vs. 29.97 Hz; Figure 3g). However, individual LHb neurons were diversely modulated by spontaneous behavioral state fluctuations: the majority of neurons (60/92, 65.2%) showed a significant increase in mean firing rates during periods of “active” behavioral state, while some exhibited reduced mean firing rates (18/92, 19.6%) or no significant modulation (14/92; 15.2%; Figure 3h; see Section 2). Notably, these findings are consistent with LHb responses to FS, where most neurons were excited, while a minority were inhibited or non‐responsive (Congiu et al. 2023; Congiu et al. 2019).

To quantify the degree of behavioral state modulation, we computed a BSMI for each neuron (see Section 2). We found that compared to other firing pattern types, Type‐1 neurons were more strongly modulated by spontaneous behavior, as indicated by a comparatively higher BSMI (ANOVA, *p* < 0.005, n neurons = 92; Type‐1 vs. Type‐2: mean difference = −0.118, 95% CI [−0.229, −0.008], *p* = 0.0306; Type‐1 vs. Type‐3: mean difference = −0.151, 95% CI [−0.259, −0.042], *p* = 0.0027; Type‐1 vs. Type‐4: mean difference = −0.180, 95% CI [−0.296, −0.063], *p* = 0.6 × 10^–3^; Figure 3i,j with results of Tukey's HSD). These results indicate that LHb neurons are significantly modulated by spontaneous behavioral state fluctuations, with Type‐1 neurons exhibiting, on average, the strongest modulation.

## 4 Discussion

In the present study, we investigated the morphological and physiological diversity of neurons in the mouse LHb, a brain region involved in processing aversive stimuli and regulating motivated behaviors. Previous work has revealed a high degree of electrophysiological and molecular diversity within LHb circuits; whether such diversity also applies to in vivo firing patterns has remained largely unexplored. By in vivo recording, labeling, and reconstructing single LHb neurons, we found a direct axonal projection from the LHb to the MD thalamus (Figure 1c,d), as suggested by tracing studies (Araki et al. 1988; Cornwall and Phillipson 1988; Herkenham and Nauta 1979; Huang et al. 2024). Within the MD, these axons exhibited extensive branching, frequently associated with large boutons (Figure 1c,d), suggesting strong postsynaptic influence. The MD is primarily involved in higher order cognitive processes, including behavioral flexibility, working memory, and decision‐making, through its dense reciprocal connectivity with the prefrontal cortex (Parnaudeau et al. 2013; Phillips et al. 2019). LHb to MD projections might thus provide a route through which motivational LHb signals could rapidly modulate prefrontal MD‐mediated computations, potentially relevant to motivational disorders and executive dysfunction.

By applying an unsupervised clustering approach to in vivo electrophysiological data, we identified four distinct spontaneous firing pattern types in LHb neurons. Notably, these firing pattern types were consistently observed in both anesthetized and awake states and irrespective of the number of electrophysiological features used for clustering or accounting for collinearity between features, indicating that this diversity might be imposed by structural determinants. Indeed, our observations are consistent with previous ex vivo studies, which identified four distinct firing pattern types in the rodent LHb (Wagner et al. 2017; Wallace et al. 2020), and transcriptomic studies, which also identified four molecularly defined LHb cell types, each with its own connectivity (Wallace et al. 2020). In our limited dataset of morphologically identified and reconstructed neurons, we did not find evidence for morphological correlates of the different in vivo firing types (Figure S4); these conclusions, however, rest on a limited dataset, and future work should explore structure–function relationships in the LHb.

We note that our physiological observations are based on unsupervised clustering of firing patterns. Hence, whether the four firing pattern types are discrete or rather map onto a continuum of neuronal features remains to be investigated. Moreover, whether the firing pattern type (i.e., cluster affiliation) of neurons is fixed (i.e., pre‐determined by, for example, intrinsic cellular properties and/or synaptic inputs) or dynamically modulated (e.g., by behavioral state and/or learning) remains to be investigated. We speculate that the diversity in firing patterns observed in vivo might correlate to the known diversity of intrinsic neuronal properties, morphologies, and input connectivity described ex vivo (Wagner et al. 2017; Weiss and Veh 2011). Indeed, the tendency of neurons to fire spike bursts and input–output connectivity (Li et al. 2011; Maroteaux and Mameli 2012; Michel et al. 2024; Shabel et al. 2012) is anatomically organized within the LHb. Future work will be required for testing this hypothesis and for resolving possible structure–function relationships within LHb circuits.

Spontaneous fluctuations in behavioral state modulate neuronal activity throughout the brain, yet only a small fraction of neurons in each brain structure are strongly modulated (Bimbard et al. 2023; Blanco‐Hernandez et al. 2024; Oude Lohuis et al. 2024; Steinmetz et al. 2019; Stringer et al. 2019). Indeed, in line with previous work, we found that only a minority of neurons within the LHb were particularly responsive to aversive stimuli in anesthetized mice and modulated by behavioral state in awake mice. Interestingly, under both anesthetized and awake conditions, these modulated neurons were preferentially recruited from Type‐1 firing patterns. Compared to the other clusters, these neurons also displayed a higher tendency to fire spike bursts. Since spike bursts have been linked to plasticity and excitability (Krahe and Gabbiani 2004; Lisman 1997), our data indicate that these minority Type‐1 LHb neurons might play a fundamental role in information encoding within the LHb. This hypothesis is indeed consistent with a large body of previous work, which demonstrated that minorities of “excitable and plastic” cells dominate information encoding and processing in different brain areas like neocortex (Yassin et al. 2010), hippocampus (Zhang and Jonas 2020), and amygdala (Feng et al. 2016). We acknowledge that the relationship between LHb activity and facial motion in our data is correlational; therefore, future work is necessary to explore the bidirectional dynamics of neural‐behavioral interactions. Neuronal spike bursts are also particularly efficient at overcoming synaptic failures and transmitting information to downstream targets (Krahe and Gabbiani 2004). Indeed, bursts in LHb neurons efficiently inhibit reward‐related downstream targets and have been shown to play an important role in diseases including depression (Yang et al. 2018). Hence, Type‐1 neurons might also play a dominant role in pathophysiological LHb states. We speculate that modulating the activity of Type‐1 neurons may be a particularly powerful approach for modulating LHb computations as demonstrated, for examplr, in the hippocampus, neocortex, and amygdala, where artificial modulation of “active minorities” has been shown to be sufficient for modifying memory content and for driving behavior (Dorst and Ramirez 2024; Josselyn and Frankland 2018; Josselyn and Tonegawa 2020). Whether Type‐1 LHb neurons can be preferentially tagged with immediate early gene expression as, for example, in cortico‐hippocampal and amygdala networks (Josselyn et al. 2015; Pang et al. 2023) remains to be investigated.

In summary, our data provide insights into the diversity of LHb in vivo firing patterns and point to low‐firing, bursty (Type‐1) neurons playing a unique role in information encoding within LHb circuits.

## Author Contributions

**Ioannis S. Zouridis:** conceptualization, data curation, formal analysis, investigation, methodology, project administration, software, validation, visualization, writing – original draft, writing – review and editing. **Lisa Schmors:** conceptualization, data curation, formal analysis, methodology, project administration, software, validation, visualization, writing – original draft, writing – review and editing. **Salvatore Lecca:** investigation, writing – review and editing. **Mauro Congiu:** investigation, writing – review and editing. **Manuel Mameli:** data curation, supervision, writing – review and editing. **Philipp Berens:** conceptualization, funding acquisition, resources, supervision, writing – review and editing. **Fabio Monteiro:** investigation. **Patricia Preston‐Ferrer:** formal analysis, investigation. **Andrea Burgalossi:** conceptualization, funding acquisition, project administration, resources, supervision, writing – review and editing.

## Conflicts of Interest

The authors declare no conflicts of interest.

## Supporting information


**Figure S1:** Electrophysiological properties of LHb neurons recorded under ketamine‐xylazine or isoflurane anesthesia. (a) t‐SNE embedding of electrophysiological properties of LHb neurons recorded under ketamine (red; n_neurons_ = 149) or isoflurane (blue; n_neurons_ = 121) anesthesia. Each point represents a neuron. (b) Comparison of mean firing rates of neurons recorded under ketamine or isoflurane anesthesia (n.s., non‐significant). (c) Comparison of coefficient of variation of neurons recorded under ketamine or isoflurane anesthesia (n.s., non‐significant). (d) Comparison of burst indices of neurons recorded under ketamine‐xylazine or isoflurane anesthesia (**, *p* < 0.01, two‐sided Wilcoxon rank‐sum test). (e) Comparison of mean autocorellograms computed for neurons recorded under ketamine‐xylazine and isoflurane anesthesia. Shaded area indicates ±1 SD. Black bars mark the time periods of statistically significant difference. (f) Same as in (e) but for interspike interval distributions. (g) Mean firing rate for each firing pattern type (1–4) under ketamine anesthesia (left) and isoflurane (right). Number of recordings and conventions as in (a). (h) Same as in (g) but for coefficient of variation and (i) for burst index. (j, k) Mean autocorrelograms (j) and interspike interval distributions (k) for LHb neurons recorded under ketamine‐xylazine (left) and isoflurane (right) anesthesia. Number of neurons and conventions as in (a). (l) Firing pattern types split by type of anesthetic. In all firing pattern types, both ketamine‐xylazine and isoflurane anesthetics are represented with at least 10 neurons (Type‐1 *n* = 15 for ketamine‐xylazine and *n* = 46 neurons for isoflurane, Type‐2 *n* = 46 neurons for ketamine‐xylazine and *n* = 32 neurons for isoflurane, Type‐3 *n* = 60 neurons for ketamine‐xylazine and *n* = 12 neurons for isoflurane, and Type‐4 *n* = 28 neurons for ketamine‐xylazine and *n* = 31 neurons for isoflurane).
**Figure S2:** Distributions of features and their correlations across LHb neurons in anesthetized and awake conditions. (a) ACG feature extraction using sparse Principal Components Analysis (sPCA). Top: Mean z‐scored ACG for all neurons (black; n_neurons_ = 565). Bottom: Weight for all eight sPCA components across time. (b) Same as in (a) but for interspike‐interval distributions. (c) Top: Bayesian information criterion (BIC) computed for different numbers of clusters and different number of sPCA components for the anesthetized (left) and awake (right) condition. Red dashed line indicates n_clusters_ = 4. For each number of clusters, the GMM was initialized randomly 100 times. Black indicates the mean across all numbers of sPCA components. Bottom: Same as in (top) but for the gradient of BIC curve. (d) Feature importance computed as the absolute difference of cluster means from the global mean for each feature separately for the condition. (e) Same as in (d) but for the awake condition. (f) Cross‐correlation between features for the anesthetized condition. (g) Same as in (f) but for the awake condition. (h) t‐SNE embedding of electrophysiological properties of LHb neurons recorded under anesthesia. Neurons are color‐coded by the respective feature, from left to right: firing rate, coefficient of variation, burst index, principle component for ISI, and principal component for ACG (only the first principle component is shown here). (i) Same as in (h) but for neurons recorded under anesthesia.
**Figure S3:** Electrophysiological properties of LHb neurons recorded under anesthesia and awake conditions. (a‐c) Distributions of firing rate (a), burst index (b), and coefficient of variation (c) of LHb neurons recorded under anesthesia (purple; n_neurons_ = 270) and awake (green; n_neurons_ = 295) conditions. (d‐e) Autocorrelograms (d) and interspike interval distributions (e) computed for LHb neurons recorded under anesthesia (purple) and awake (green) conditions. Lines indicate the mean, shaded areas indicate ±1 SD. (f) t‐SNE embedding of electrophysiological properties of LHb neurons recorded under anesthesia (purple; n_neurons_ = 270) and awake conditions (green; n_neurons_ = 295). Each point represents a recording.
**Figure S4:** Topographical distribution and primary dendritic features of LHb neurons according to their firing pattern type. (a) Schematic representation of the partitioning of LHb in four quartiles, namely dorsomedial (DM), dorsolateral (DL), ventromedial (VM), and ventrolateral (VL). Anatomical orientation: Dorsal, up; lateral, left. LHb, magenta; Medial habenula, green. (b) Topographical distribution of firing patterns in quartiles (“n” refers to the subset of LHb neurons which were morphologically identified, that is, referring to recordings that could be unequivocally matched to neurons successfully recovered and localized within the LHb). Firing pattern Type‐1, dorsolateral (DL): 1/6, dorsomedial (DM): 0/6, ventrolateral (VL): 3/6, ventromedial (VM): 2/6; Firing pattern Type‐2: DL: 2/16, DM: 4/16, VL: 4/16, VM: 6/16; Firing pattern Type‐3: DL: 5/29, DM: 2/29, VL: 18/29, VM: 4/29; Type‐4: DL: 1/12, DM: 2/12, VL: 5/12, VM: 4/12. (c) t‐SNE embedding based on spontaneous firing features of LHb neurons recorded under anesthesia (each point represents a recording; n_neurons_ = 270). Firing pattern types (color‐coded) were determined using Gaussian Mixture Modelling (GMM) clustering. Morphologically reconstructed neurons are indicated as black circles with n_Type‐1_ = 6/61 (morphologically‐reconstructed vs. all Type‐1 neurons), n_Type‐2_ = 9/78, n_Type‐3_ = 12/72, n_Type‐4_ = 3/59 (“n” refers to the subset of LHb neurons which were morphologically reconstructed). (d) Number of primary dendrites for each firing pattern type. “n” (morphologically‐reconstructed neurons) and conventions as in (c). (e) Same as in (d) but for the total dendritic length. (f) Same as in (d) but for the total number of dendritic nodes. (g) Same as in (d) but for the maximum order of dendrites.

## Data Availability

All code to generate results and figures is available at https://github.com/berenslab/cell_types_lateral_habenula.git and the data can be downloaded under https://doi.org/10.5281/zenodo.16876960.
