# Supplementary material for: Aversion Encoding and Behavioral State Modulation of Physiologically Defined Cell Types in the Lateral Habenula
Source: Eur J Neurosci. 2025 Nov 24;62(10):e70302. doi: 10.1111/ejn.70302 (PMC12643828; doi:10.1111/ejn.70302)
Supplement: Supplementary file 1 — Figure S1: Electrophysiological properties of LHb neurons recorded under ketamine‐xylazine or isoflurane anesthesia. (a) t‐SNE embedding of electrophysiological properties of LHb neurons recorded under ketamine (red; nneurons = 149) or isoflurane (blue; nneurons = 121) anesthesia. Each point represents a neuron. (b) Comparison of mean firing rates of neurons recorded under ketamine or isoflurane anesthesia (n.s., non‐significant). (c) Comparison of coefficient of variation of neurons recorded under ketamine or isoflurane anesthesia (n.s., non‐significant). (d) Comparison of burst indices of neurons recorded under ketamine‐xylazine or isoflurane anesthesia (**, p < 0.01, two‐sided Wilcoxon rank‐sum test). (e) Comparison of mean autocorellograms computed for neurons recorded under ketamine‐xylazine and isoflurane anesthesia. Shaded area indicates ±1 SD. Black bars mark the time periods of statistically significant difference. (f) Same as in (e) but for interspike interval distributions. (g) Mean firing rate for each firing pattern type (1–4) under ketamine anesthesia (left) and isoflurane (right). Number of recordings and conventions as in (a). (h) Same as in (g) but for coefficient of variation and (i) for burst index. (j, k) Mean autocorrelograms (j) and interspike interval distributions (k) for LHb neurons recorded under ketamine‐xylazine (left) and isoflurane (right) anesthesia. Number of neurons and conventions as in (a). (l) Firing pattern types split by type of anesthetic. In all firing pattern types, both ketamine‐xylazine and isoflurane anesthetics are represented with at least 10 neurons (Type‐1 n = 15 for ketamine‐xylazine and n = 46 neurons for isoflurane, Type‐2 n = 46 neurons for ketamine‐xylazine and n = 32 neurons for isoflurane, Type‐3 n = 60 neurons for ketamine‐xylazine and n = 12 neurons for isoflurane, and Type‐4 n = 28 neurons for ketamine‐xylazine and n = 31 neurons for isoflurane). Figure S2: Distributions of features and their correlations acr [file EJN-62-0-s001.pdf]

Supporting Information

Aversion encoding and behavioral state modulation of lateral habenula neurons

Ioannis S. Zouridis, Lisa Schmors, Salvatore Lecca, Mauro Congiu, Manuel Mameli, Philipp Berens, Fabio Monteiro, Patricia Preston-Ferrer, Andrea Burgalossi

Figure S1

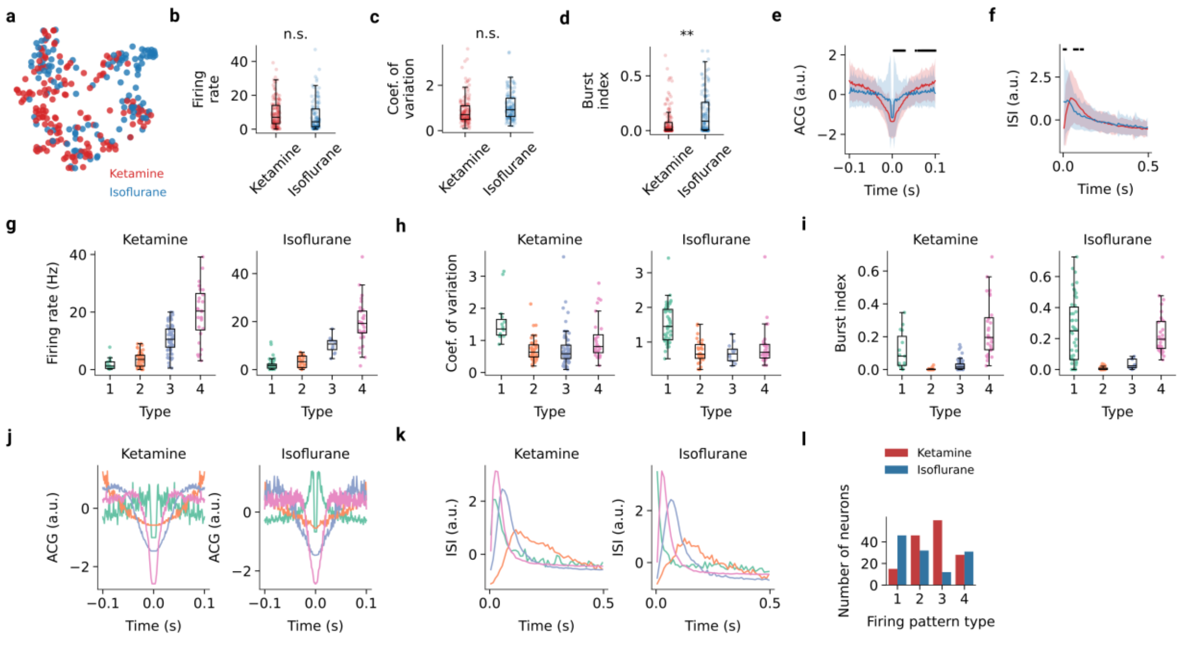

Figure S1: Electrophysiological properties of LHb neurons recorded under ketamine-xylazine or isoflurane anesthesia.

(a) t-SNE embedding of electrophysiological properties of LHb neurons recorded under ketamine (red; $n_{neurons}$=149) or isoflurane (blue; $n_{neurons}$=121) anesthesia. Each point represents a neuron. (b) Comparison of mean firing rates of neurons recorded under ketamine or isoflurane anesthesia (n.s., non-significant). (c) Comparison of coefficient of variation of neurons recorded under ketamine or isoflurane anesthesia (n.s., non-significant). (d) Comparison of burst indices of neurons recorded under ketamine-xylazine or isoflurane anesthesia (∗∗, $p < 0.01$, two-sided Wilcoxon rank-sum test). (e) Comparison of mean autocorellograms computed for neurons recorded under ketamine-xylazine and isoflurane anesthesia. Shaded area indicates ±1 SD. Black bars mark the time periods of statistically significant difference. (f) Same as in (e) but for interspike interval distributions (g) Mean firing rate for each firing pattern type (1-4) under ketamine anesthesia (left) and isoflurane (right). Number of recordings and conventions as in (a). (h) Same as in (g) but for coefficient of variation and (i) for burst index. (j, k) Mean autocorrelograms (j) and interspike interval distributions (k) for LHb neurons recorded under ketamine-xylazine (left) and isoflurane (right) anesthesia. Number of neurons and conventions as in (a). (l) Firing pattern types split by type of anesthetic. In all firing pattern types, both ketamine-xylazine and isoflurane anesthetics are represented with at least 10 neurons (Type-1 n=15 for ketamine-xylazine and n=46 neurons for isoflurane, Type-2 n=46 neurons for ketamine-xylazine and n=32 neurons for isoflurane, Type-3 n=60 neurons for ketamine-xylazine and n=12 neurons for isoflurane, and Type-4 n=28 neurons for ketamine-xylazine and n=31 neurons for isoflurane).

Figure S2

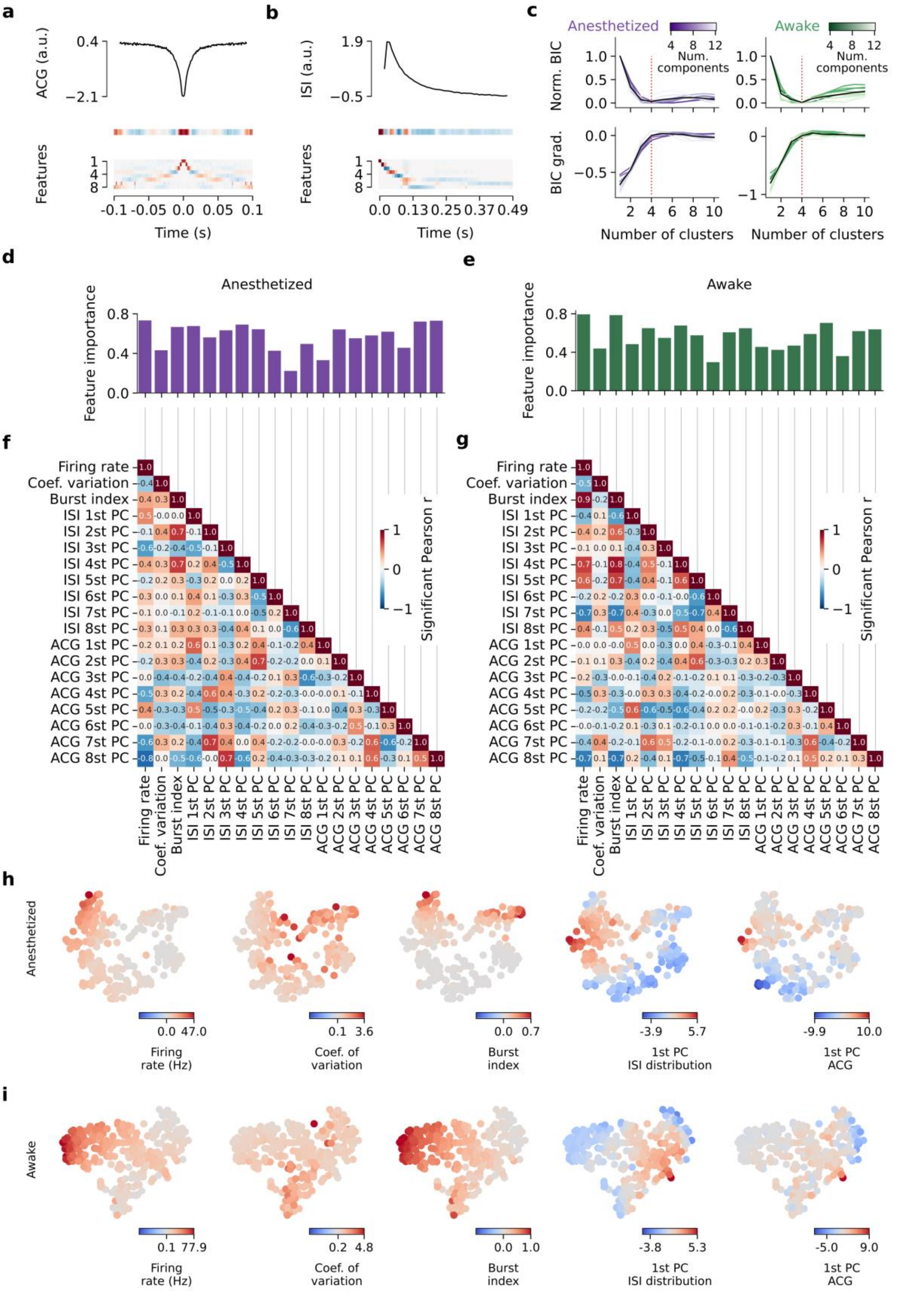

Figure S2: Distributions of features and their correlations across LHb neurons in anesthetized and awake conditions.

(a) ACG feature extraction using sparse Principal Components Analysis (sPCA). Top: Mean z-scored ACG for all neurons (black; $n_{neurons}$=565). Bottom: Weight for all 8 sPCA components across time. (b) Same as in (a) but for interspike-interval distributions. (c) Top: Bayesian information criterion (BIC) computed for different numbers of clusters and different number of sPCA components for the anesthetized (left) and awake (right) condition. Red dashed line indicates $n_{clusters}$=4. For each number of clusters, the GMM was initialized randomly 100 times. Black indicates the mean across all numbers of sPCA components. Bottom: Same as in (top) but for the gradient of BIC curve. (d) Feature importance computed as the absolute difference of cluster means from the global mean for each feature separately for the anesthetized condition. (e) Same as in (d) but for the awake condition. (f) Cross-correlation between features for the anesthetized condition. (g) Same as in (f) but for the awake condition. (h) t-SNE embedding of electrophysiological properties of LHb neurons recorded under anesthesia. Neurons are color-coded by the respective feature, from left to right: firing rate, coefficient of variation, burst index, principle component for ISI, and principal component for ACG (only the first principle component is shown here). (i) Same as in (h) but for neurons recorded under anesthesia.

Figure S3

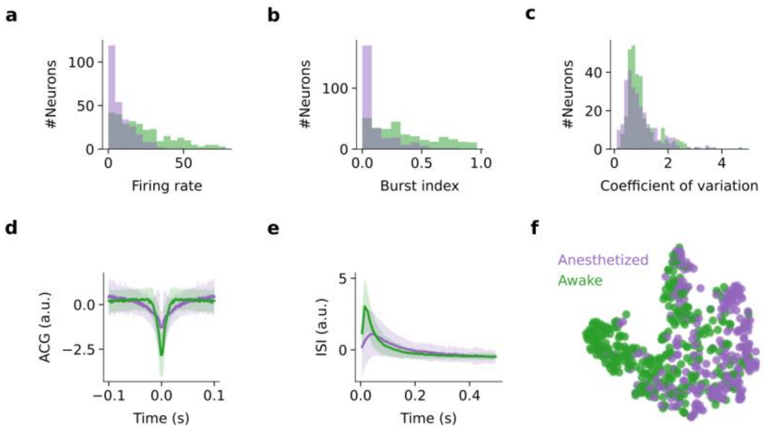

Figure S3: Electrophysiological properties of LHb neurons recorded under anesthesia and awake conditions.

(a-c) Distributions of firing rate (a), burst index (b), and coefficient of variation (c) of LHb neurons recorded under anesthesia (purple; $n_{neurons}$=270) and awake (green; $n_{neurons}$=295) conditions. (d-e) Autocorrelograms (d) and interspike interval distributions (e) computed for LHb neurons recorded under anesthesia (purple) and awake (green) conditions. Lines indicate the mean, shaded areas indicate ±1 SD. (f) t-SNE embedding of electrophysiological properties of LHb neurons recorded under anesthesia (purple; $n_{neurons}$=270) and awake conditions (green; $n_{neurons}$=295). Each point represents a recording.

Figure S4

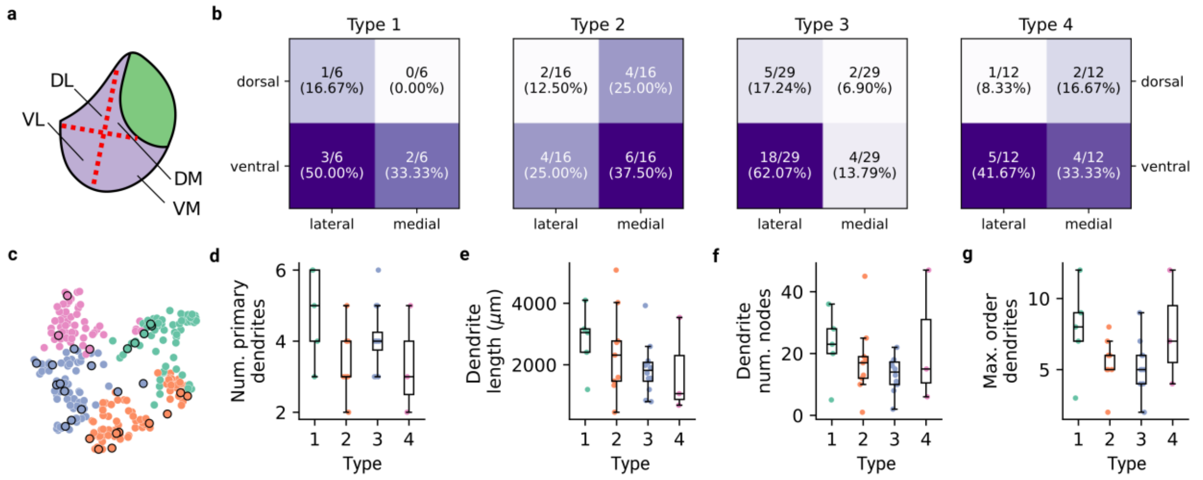

Figure S4: Topographical distribution and primary dendritic features of LHb neurons according to their firing pattern type.

(a) Schematic representation of the partitioning of LHb in four quartiles, namely dorsomedial (DM), dorsolateral (DL), ventromedial (VM), and ventrolateral (VL). Anatomical orientation: Dorsal, up; lateral, left. LHb, magenta; Medial habenula, green. (b) Topographical distribution of firing patterns in quartiles ('n' refers to the subset of LHb neurons which were *morphologically identified* i.e. referring to recordings that could be unequivocally matched to neurons successfully recovered and localized within the LHb). Firing pattern Type-1, dorsolateral (DL): 1/6, dorsomedial (DM): 0/6, ventrolateral (VL): 3/6, ventromedial (VM): 2/6; Firing pattern Type-2: DL: 2/16, DM: 4/16, VL: 4/16, VM: 6/16; Firing pattern Type-3: DL: 5/29, DM: 2/29, VL: 18/29, VM: 4/29; Type-4: DL: 1/12, DM: 2/12, VL: 5/12, VM: 4/12. (c) t-SNE embedding based on spontaneous firing features of LHb neurons recorded under anesthesia (each point represents a recording; $n_{neurons}$=270). Firing pattern types (color-coded) were determined using Gaussian Mixture Modeling (GMM) clustering. Morphologically reconstructed neurons are indicated as black circles with n_{Type-1}=6/61 (morphologically-reconstructed vs all Type-1 neurons), n_{Type-2}=9/78, n_{Type-3}=12/72, n_{Type-4}=3/59 ('n' refers to the subset of LHb neurons which were *morphologically reconstructed*). (d) Number of primary dendrites for each firing pattern type. 'n' (morphologically-reconstructed neurons) and conventions as in (c). (e) Same as in (d) but for the total dendritic length. (f) Same as in (d) but for the total number of dendritic nodes. (g) Same as in (d) but for the maximum order of dendrites.
